# Involvement of Abnormal Gut Microbiota Composition and Function in Doxorubicin-Induced Cardiotoxicity

**DOI:** 10.3389/fcimb.2022.808837

**Published:** 2022-02-25

**Authors:** Jie Huang, Shanshan Wei, Chuanhao Jiang, Zijun Xiao, Jian Liu, Weijun Peng, Bikui Zhang, Wenqun Li

**Affiliations:** ^1^ Department of Pharmacy, The Second Xiangya Hospital, Central South University, Changsha, China; ^2^ Institute of Clinical Pharmacy, Central South University, Changsha, China; ^3^ Department of Laboratory Medicine, The Second Xiangya Hospital, Central South University, Changsha, China; ^4^ Department of Integrated Traditional Chinese & Western Medicine, The Second Xiangya Hospital, Central South University, Changsha, China

**Keywords:** doxorubicin, cardiotoxicity, gut microbiota, 16S rRNA gene sequencing, metagenomic sequencing

## Abstract

**Objectives:**

Doxorubicin (Dox), a chemotherapeutic anthracycline agent for the treatment of a variety of malignancies, has a limitation in clinical application for dose-dependent cardiotoxicity. The purpose of this study was to explore the relationship between the composition/function of the gut microbiota and Dox-induced cardiotoxicity (DIC).

**Methods:**

C57BL/6J mice were injected intraperitoneally with 15 mg/kg of Dox, with or without antibiotics (Abs) administration. The M-mode echocardiograms were performed to assess cardiac function. The histopathological analysis was conducted by H&E staining and TUNEL kit assay. The serum levels of creatine kinase (CK), CK-MB (CK-MB), lactic dehydrogenase (LDH), and cardiac troponin T (cTnT) were analyzed by an automatic biochemical analyzer. 16S rRNA gene and metagenomic sequencing of fecal samples were used to explore the gut microbiota composition and function.

**Key Findings:**

Dox caused left ventricular (LV) dilation and reduced LV contractility. The levels of cardiomyocyte apoptosis and myocardial enzymes were elevated in Dox-treated mice compared with the control (Con) group. 16S rRNA gene sequencing results revealed significant differences in microbial composition between the two groups. In the Dox group, the relative abundances of *Allobaculum*, *Muribaculum*, and *Lachnoclostridium* were significantly decreased, whereas *Faecalibaculum*, *Dubosiella*, and Lachnospiraceae were significantly increased compared with the Con group at the genus level. Functional enrichment with Cluster of orthologous groups of proteins (COG) and Kyoto Encyclopedia of Genes and Genomes (KEGG) analyses showed that the Dox mice displayed different clusters of cellular processes and metabolism from the Con mice. The different species and their functions between the two groups were associated with the clinical factors of cardiac enzymes. Moreover, depletion of the gut microbiota could alleviate Dox-induced myocardial injury and cardiomyocyte apoptosis.

**Conclusions:**

The study here shows that composition imbalance and functional changes of the gut microbiota can be one of the etiological mechanisms underlying DIC. The gut microbiota may serve as new targets for the treatment of cardiotoxicity and cardiovascular diseases.

## Introduction

Doxorubicin (Dox) is a chemotherapeutic anthracycline agent with broad-spectrum and high efficacy used for the treatment of a variety of malignancies (Wu et al., 2021). However, its clinical application is limited due to the dose-dependent cardiotoxicity, which may lead to acute pericarditis, irreversible cardiomyopathy, and congestive heart failure (HF) (Wenningmann et al., 2019). The pathogenesis of Dox may be associated with topoisomerase IIβ (Top2b) inhibition, oxidative stress, inflammation, and apoptosis (Liu et al., 2020a; Tadokoro et al., 2020). Though a variety of strategies including limitation of cumulative Dox doses, use of antioxidant drugs, and common HF drugs have been proposed to prevent or attenuate Dox-induced cardiotoxicity (DIC), none of these strategies have obtained satisfying efficacy (Vejpongsa and Yeh, 2014; Varricchi et al., 2018; Yarmohammadi et al., 2021). Given that the mechanism of DIC is a complex disturbance system, an alternative hypothesis is required to explain DIC, and a novel therapeutic strategy needs to be established.

From the outcome of recent studies, the intestinal microbiota has been found to play an essential role in the health of the host organism. Multiple diseases, such as diabetes, obesity, cancer, and nervous system disease, are related to the changes of intestinal microorganisms (Cheng et al., 2020; Fan and Pedersen, 2020; Megur et al., 2020; Verhaar et al., 2020). Particularly, the gut microbiota and their metabolites have been implicated in the progression of cardiovascular diseases (CVDs) including hypertension, dyslipidemia, atherosclerosis, thrombosis, HF, and ischemic stroke (Kasahara and Rey, 2019). In addition, it has become evident that the gut microbiota affects the response to cancer therapy and susceptibility to toxic side effects (Roy and Trinchieri, 2017). Besides, drugs can alter the microbiome and create secondary effects independent of the drug molecule itself (Hitchings and Kelly, 2019). Recently, antitumor chemotherapy drugs, such as cisplatin and Dox, have been reported to cause alteration of the gut microbiota, which is involved in the pathogenesis of cardiotoxicity (Zhao et al., 2018; Wu et al., 2019). However, previous studies were based on a 16S rRNA gene sequencing method to focus on changes in microbial composition, which has the limitations such as the bias of PCR amplification (Silverman et al., 2021) and difficulty to identify most microbes at the strain and species levels (Peng et al., 2018). Compared to the amplicon, the shotgun metagenome can provide functional gene profiles directly and reach a much higher resolution of taxonomic annotation (Liu et al., 2021). Therefore, further studies conducting metagenome sequencing are required to analyze the functional activity of the gut microbiome linked with DIC.

Here, to investigate the key microbes related to cardiotoxicity and microbial functions from fecal samples, we constructed an explicit mouse model of DIC. Using a combination of 16S rRNA gene sequencing and metagenomic sequencing analysis, we strived to compare the composition and function of the gut microbiome between the normal control (Con) mice and Dox mice. We also conducted an antibiotic intervention in mice to further understand the role of the gut microbiome in DIC.

## Materials and Methods

### Drugs and Reagents

Dox for injection was obtained from Shenzhen Main Luck Pharmaceuticals Co., Ltd. (Shenzhen, China). Four antibiotics (Abs) including vancomycin, metronidazole, ampicillin, and neomycin were purchased from Zhengde Pharmaceutical Co., Ltd. (Taiwan, China), Huazhong Pharmaceutical Co., Ltd. (Xiangyang, China), Hunan Kangerjia Biomedical Technology Co., Ltd. (Zhangjiajie, China), and BBI Life Sciences Co., Ltd. (Shanghai, China), respectively.

### Animals and Treatments

Female C57BL/6J mice aged 6–8 weeks were obtained from Laboratory Animal Center, Xiangya School of Medicine, Central South University (Changsha, China). All the procedures in this research were operated in accordance with the National Institutes of Health Guide (NIH publications no. 8023) for the Care and Use of Laboratory Animals. The experimental protocol was approved by the Medicine Animal Welfare Committee of Xiangya School of Medicine (SYXK-2015/0017).

To establish the cardiotoxicity model, the mice were randomly divided into two groups with 10 animals in each group, including the Con group and Dox group. The Dox group (15 mg/kg) was administrated with 3 mg/kg every other day by intraperitoneal injection. For antibiotic treatment, the mice were randomly divided into three groups including the Con group, Dox group, and Dox+Abs group. The Dox+Abs group was treated with drinking water containing 500 mg/L of each antibiotic for 28 days. Dox was injected intraperitoneally after 14 days of Abs. On the 28th day, the mice were subjected to echocardiography for evaluating cardiac function. Besides, the fecal samples from the Con group and Dox group were collected into sterile cryopreservation tubes, frozen quickly in liquid nitrogen, and then refrigerated at −80°C for DNA extraction and amplification of the 16S rRNA gene. Subsequently, all animals were anesthetized with 1% pentobarbital sodium (50 mg/kg, i.p.). The venous blood was drawn from the orbit of mice. The heart tissues were acquired after perfusing and rinsing with cold saline and then preserved in 4% paraformaldehyde for histopathological analysis.

### M-Mode Echocardiograms

M-mode echocardiograms were performed by a VisualSonics Vevo 2100 (VisualSonics, Toronto, ON, Canada). The mice were anesthetized with 1.5% isoflurane, and an appropriate amount of coupling agent was applied to the left anterior chest area after hair removal. Then the M-ultrasound changes of 10~20 cardiac cycles were recorded. Finally, the parameters of cardiac function including left ventricular (LV) ejection fraction (EF%) and LV fractional shortening (FS%) were calculated.

### Histopathological Analysis

After being fixed in 4% paraformaldehyde at room temperature, the heart tissues were embedded in paraffin wax and cut into 3-μm thin sections. After being dehydrated in a series of graded alcohols, the sections were stained with H&E and TdT-mediated dUTP Nick-End Labeling (TUNEL) kit assay according to the manufacturer’s instructions.

### Biochemical Analysis

After Abs and Dox treatment, the collected blood samples were centrifuged at 3,000 rpm at 4°C for 15 min to obtain serum. The serum biochemical parameters including creatine kinase (CK), CK-MB (CK-MB), lactic dehydrogenase (LDH), and cardiac troponin T (cTnT) were analyzed by using kits with an automatic biochemical analyzer according to the manufacturers’ instructions.

### 16S rRNA Gene Sequence Analysis

Fecal bacterial DNA was extracted using the E.Z.N.A.^®^ Stool DNA Kit (Omega Biotech, Norcross, GA, USA) according to the manufacturer’s protocol. The extracted genomic DNA was detected by 1% agarose gel electrophoresis. To assess bacterial diversity, the V3–V4 hypervariable regions of the bacterial 16S rRNA gene were amplified with a set of primers (338F 5′ACTCCTACGGGAGGCAGCAG-3′, 806R 5′GGACTACHVGGGTWTCTAAT-3′). Then, the PCR products were detected using 2% agarose gel electrophoresis, recovered using AxyPrepDNA Gel Recovery Kit (Axygen Biosciences, Union City, CA, USA), and quantified using QuantiFluor™-ST (Promega, Madison, WI, USA) according to the quantitative results of electrophoresis. Subsequently, purified amplicons were mixed in appropriate proportions and paired-end sequenced on an Illumina MiSeq platform (Illumina, San Diego, CA, USA) according to the standard protocols by Majorbio Bio-Pharm Technology Co. Ltd. (Shanghai, China).

Raw fastq files were demultiplexed and quality-filtered by QIIME (version 1.9.1, http://qiime.org/) and spliced by FLASH. Trimmed sequences were further clustered into operational taxonomic units (OTUs) with 97% similarity cutoffs using UPARSE (version 7.1, http://drive5.com/uparse/), and chimera filtering was performed by UCHIME.

OTUs were assigned to the closest taxonomic neighbors and relative bacterial species by the RDP Classifier algorithm (http://rdp.cme.msu.edu/) using a confidence threshold of 70%. The species alignment database of 16S bacteria is the Silva database (https://www.arb-silva.de/). The relative abundance of each taxonomic level was calculated using the QIIME tool. The indices of alpha diversity including Chao, Shannon, and Ace were analyzed by MOTHUR (version 1.30.2, https://www.mothur.org/). Principal coordinate analysis (PCoA) projections were used to describe the beta diversity.

### Metagenome Sequence Analysis

We also selected a subset of specimens (6 from the Con group and 6 from the Dox group) for metagenome sequencing following 16S rRNA gene sequence analysis. In brief, the extracted genomic DNA was detected by 1% agarose gel electrophoresis and fragmented to the appropriate length (approximately 400 bp) by using Covaris M220 (Gene Company Limited, Shanghai, China). The paired-end library was constructed by the NEXTFLEX™ Rapid DNA-Seq Kit (Bioo Scientific, Austin, TX, USA) according to the manufacturer’s protocols. Then, the blunt-ends of fragments were ligated to adapters. Hiseq X sequencing systems (Illumina Inc., San Diego, CA, USA) were used for metagenomic sequencing. The raw data were trimmed and decontaminated to obtain optimized reads, which were assembled using MEGAHIT (http://www.l3-bioinfo.com/products/megahit.htm). The assembled contigs not less than 100 bp were used for further gene prediction and annotation.

Subsequently, the open reading frames (ORFs) of the assembled sequences were predicted by using the MetaGene platform (http://metagene.cb.k.u-tokyo.ac.jp/). The redundant genes were filtered out to construct non-redundant gene sets. Genes with sequence identity greater than 95% were clustered together using CD-HIT (http://www.bioinformatics.org/cd-hit/), and the longest sequences from each cluster were the representative gene. The genetic functions were annotated and classified by the functional databases. Cluster of orthologous groups of proteins (COG) annotation was conducted by using BLASTP (version 2.3.0) against the eggNOG database (version 4.5.1, http://eggnogdb.embl.de/#/app/home) with an e-value cutoff of 1e−5. Kyoto Encyclopedia of Genes and Genomes (KEGG) pathway annotation was conducted by using BLASTP (version 2.3.0) against the KEGG database (http://www.genome.jp/kegg/) with an e-value cutoff of 1e−5.

The discrimination in COG and KEGG categories between the Con and Dox groups was identified by using linear discriminant analysis (LDA) effect size (LEfSe; http://huttenhower.sph.harvard.edu/galaxy/root?tool_id=lefse_upload). Only LDA values >2.0 at a *p*-value <0.05 were considered to be significantly enriched.

### Statistical Analysis

The data are expressed as mean ± SEM. Unpaired Student’s t-test for two comparisons or ANOVA followed by the Student–Newman–Keuls test for multiple comparisons was conducted to perform statistical analysis. A *p*-value less than 0.05 was considered statistically significant.

## Results

### Myocardial Injury and Cardiomyocyte Apoptosis Induced by Doxorubicin

**Figure 1:**
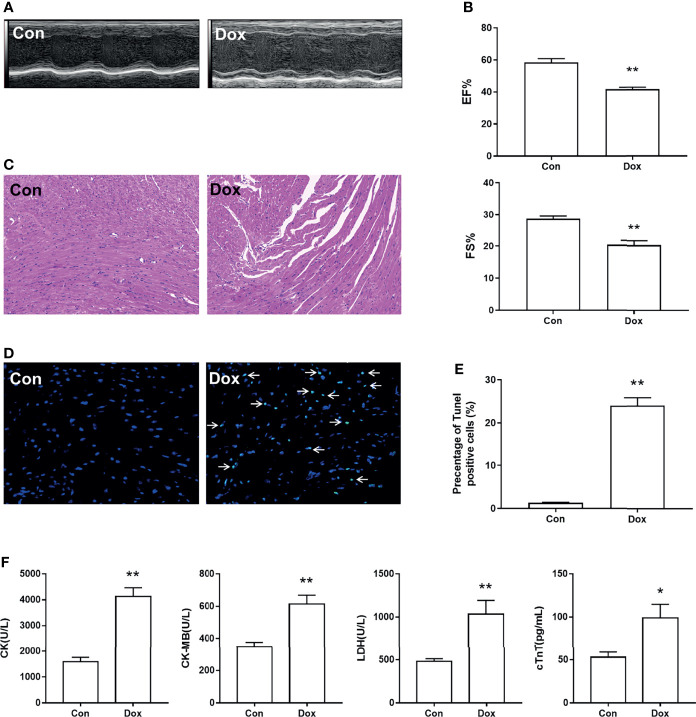
Dox-induced cardiac dysfunction and histological injury. **(A)** M-mode echocardiograms showing left ventricular dilation induced by Dox. **(B)** The parameters of left ventricular ejection fraction (EF%) and left ventricular fractional shortening (FS%). **(C)** H&E staining reflected the histological changes, magnification ×200. **(D)** Representative image of TUNEL staining, magnification ×400; white arrows indicate the apoptotic cells. **(E)** Statistical result of TUNEL staining. **(F)** The serum level of creatine kinase (CK), creatine kinase-MB (CK-MB), lactic dehydrogenase (LDH), and cardiac troponin T (cTnT). Con, control; Dox, doxorubicin. Data are mean ± SEM. n = 6–10. ^*^ *p* < 0.05, ^**^ *p* < 0.01 *vs.* Con.

First, we constructed the heart injury model induced by Dox in mice. After treatment with Dox, the M-mode echocardiograms showed LV dilation (**Figure 1A**). The EF% and FS% of the Dox group were decreased as compared with the Con group, indicating that Dox reduced LV contractility in mice (**Figure 1B**). To further determine the histological changes of the heart induced by Dox, H&E staining and TUNEL staining were conducted. As shown in **Figure 1C**, we observed regular cell distribution and normal morphology in the myocardium of the Con group. However, the Dox-treated group showed inflammatory cell infiltration, myocardial fragmentation, and disorder of cardiac fiber arrangement. TUNEL assay was used to assess the cardiomyocyte apoptosis, which is an important event in the process of DIC. The results revealed that mice with Dox treatment showed obvious cardiomyocyte apoptosis as compared with the Con group (**Figures 1D, E**). Myocardial injury was also determined by the serum levels of CK, CK-MB, LDH, and cTnT, and all of these myocardial enzymes were elevated in Dox-treated mice (**Figure 1F**). The above results suggested that Dox caused severe myocardial lesions.

### Similar Gut Microbial Diversity Between Control and Doxorubicin Mice

**Figure 2:**
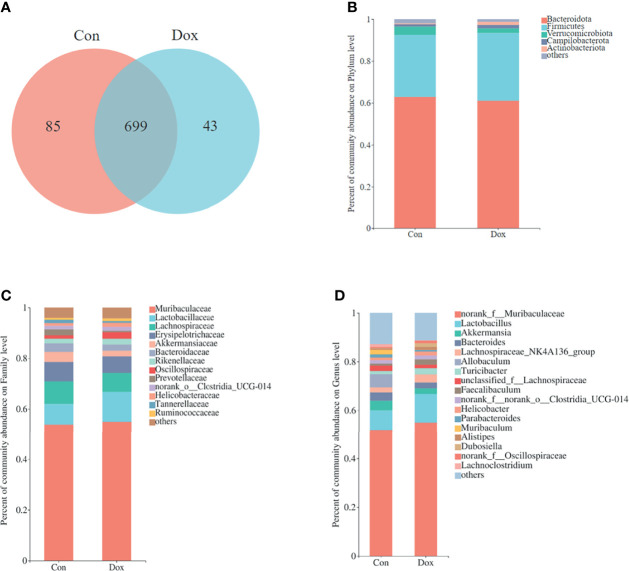
Comparison of the microbial composition between the two groups. **(A)** Venn diagram depicting OTU richness and the overlap representing the shared OTUs in microbial communities. **(B–D)** Relative abundance of microbial community for each group at phylum, family, and genus levels. Con, control; Dox, doxorubicin; OTU, operational taxonomic unit. n = 10.

To characterize the effect of Dox on gut microbial communities, we initially used 16S rRNA gene sequencing. After size filtering, quality control, and chimera removal, a total of 968,758 high-quality reads, ranging from 39,221 to 61,778 per sample, with an average length of 421.05 bp (421.05 ± 1.35 bp), were obtained from fecal samples of mice. These reads were matched into 827 OTUs (defined based on 97% sequence similarity) including 10 phyla, 272 species, and 165 genera of gut microbes. A Venn diagram showed that the two groups shared 699 OTUs, whereas 85 and 43 OTUs were unique to the Con and Dox mice, respectively (**Figure 2A**).

The relative abundance in the two groups on phylum, family, and genus levels is displayed in the bar plot (**Figures 2B–D**). The results showed that the gut microbiome compositions of the Con and Dox mice were different. The phyla Bacteroidota, Firmicutes, and Verrucomicrobiota were predominant in the gut microbiota of mice. Muribaculaceae and Lactobacillaceae were the most abundant families in fecal samples from both the Con and Dox mice, but group Dox showed an upward trend compared with group Con.

The microbial alpha diversity indices, including microbial community richness (Chao and Ace) and diversity (Shannon), were used to illustrate the changes in the microbiota community structure. All *p*-values >0.05 (Wilcoxon rank-sum test) excluding Chao (*p* = 0.0392) at the phylum level (**Supplementary Table 1**), which showed almost no significant differences between the Con and Dox groups.

### Significant Differences in the Microbial Composition Between the Two Groups

**Figure 3:**
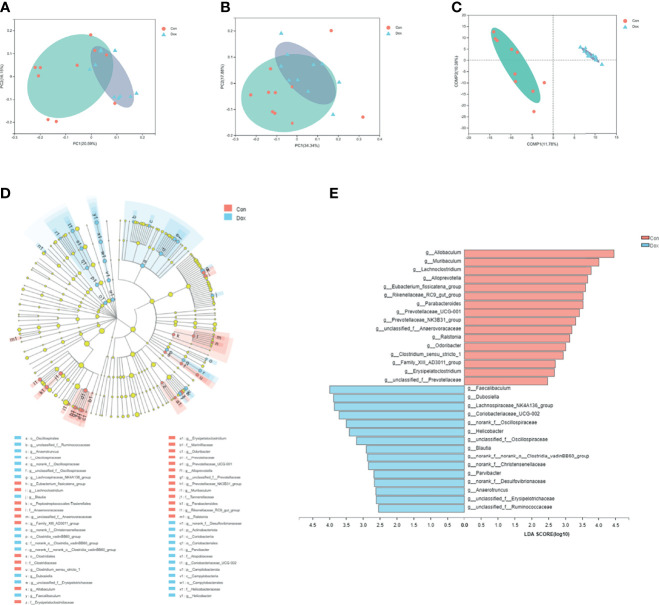
Difference analysis of gut microbial composition between the two groups. **(A, B)** Principal coordinate analysis of unweighted (R = 0.3811, *p* = 0.001000) and weighted UniFrac (R = 0.2420, *p* = 0.004000) distances. PC1 and PC2 represent the top two principal coordinates that capture the maximum diversity. **(C)** Partial least squares discriminant analysis (PLS-DA). COMP 1 and COMP 2 represent the suspected influencing factors for the deviation of the microbial composition. **(D)** Cladogram analyzed by LEfSe (LDA > 2.0, *p* < 0.05) showing the phylogenetic distribution of the bacterial lineages. Circles indicate phylogenetic levels from phylum to genus. Nodes with different colors indicate microbial taxa that are enriched in the corresponding groups and have significant differences between groups; yellow nodes indicate microbial taxa that have no significant differences between groups. The diameter of each node is proportional to the abundance of the group. **(E)** Histogram of LEfSe analysis (LDA > 2.0, *p* < 0.05) showing the LDA scores for differentially abundant genera. Con, control; Dox, doxorubicin; LDA, linear discriminant analysis; LEfSe, linear discriminant analysis effect size. n = 10.

The beta diversity analysis was carried out to reveal the difference in the microbial composition between the Con and Dox samples. As shown in PCoA, the first two principal coordinates explained 36.74% and 52.22% of the total variance for unweighted (analysis of similarities (ANOSIM) R = 0.3811, *p* = 0.001) and weighted (ANOSIM R = 0.2420, *p* = 0.004) UniFrac, respectively (**Figures 3A, B**). The partial least squares discriminant analysis (PLS-DA) showed that the bacterial communities of the two groups clustered separately (**Figure 3C**). Thus, Dox-treated mice possessed an obvious difference in a distinct clustering of fecal microbial structure as compared to the Con mice.

To further identify the significant difference in specific bacterial taxa between the Con and Dox groups, the LEfSe analysis based on discriminative features cladogram and histogram was performed, and the effect size cutoff of the LDA score was set to 2.0. This analysis identified two phyla including p_Actinobacteriota and p_Campilobacterota, and 31 genera, which were responsible for this discrimination (**Figure 3D**). In the Dox group, the relative abundances of g_*Allobaculum*, g_*Muribaculum*, and g_*Lachnoclostridium* were significantly decreased, whereas g_*Faecalibaculum*, g_*Dubosiella*, and g_Lachnospiraceae_NK4A136_group were significantly increased as compared with the Con group at the genus level (**Figure 3E**).

### The Microbial Correlation Networks Between the Two Groups Were Different

**Figure 4:**
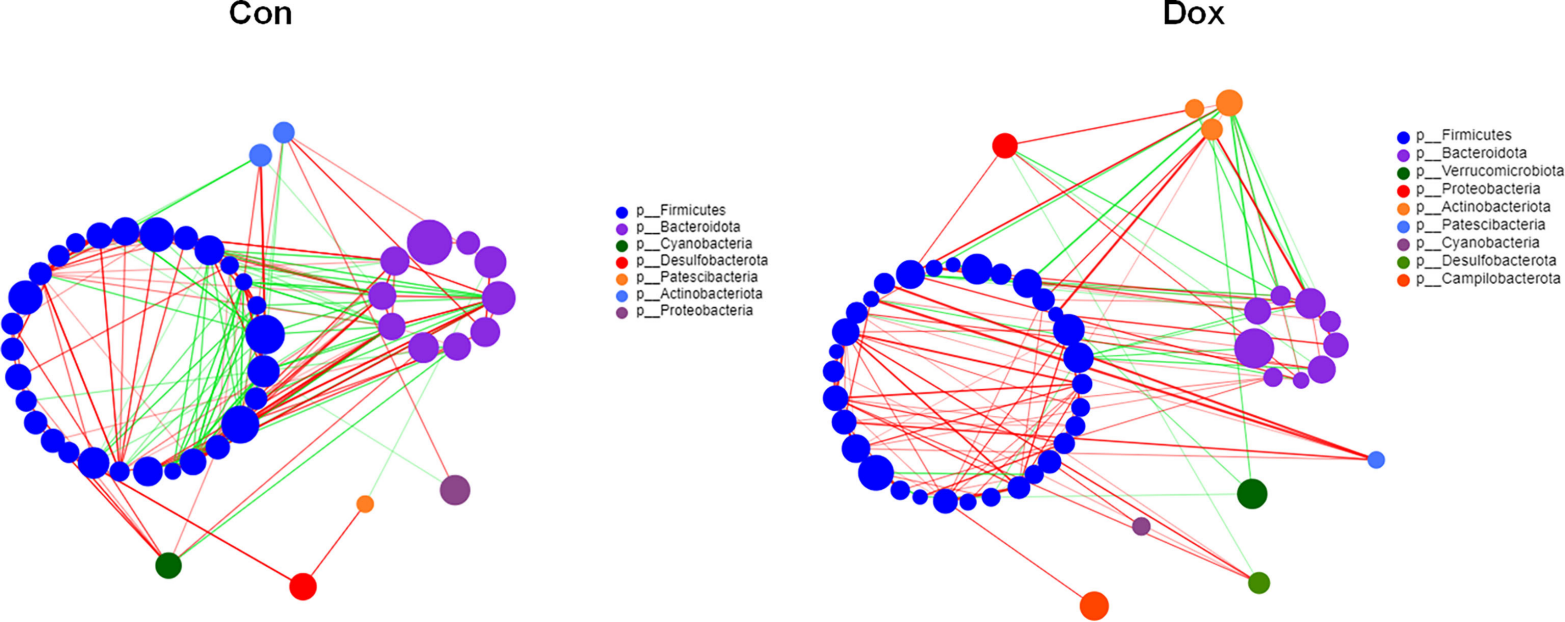
Correlation network analysis of the 50 most abundant genera for each group. The size of the node indicates the genera abundance, and different colors indicate different phyla. The lines indicate significant positive (red) and negative (green) pairwise correlations, and the thickness indicates the strength of the correlation between two genera. Spearman’s value ≥0.05, *p* < 0.05. Con, control; Dox, doxorubicin. n = 10.

To investigate the microbial correlation network, we calculated Spearman’s correlations among the 50 most abundant bacterial genera from each group. As shown in **Figure 4**, the Dox group featured more phyla (7 *vs.* 9) and displayed a stronger positive correlation among genera. The microbial community of the Dox group featured a more complicated network. In addition, the network constructed from the Dox group displayed fewer edges (334 *vs.* 125) (**Supplementary Table 2**) and lower transitivity (0.5650 *vs.* 0.4127), suggesting that the correlation among the microbiota in the Dox group was distinctly decreased compared to that of Con group. Moreover, we computed degree (DC), closeness (CC), and betweenness (BC) centrality to evaluate the taxa importance at the genus level within the network. According to the total scores of these coefficients (**Supplementary Table 3**), the top three nodes from each group were selected as putative keystone genera within this network (g_Prevotellaceae_NK3B31_group, g_Alistipes, and g_*Allobaculum* for the Con group and g_norank_f_Oscillospiraceae, g_*Bacteroides*, and g_*Faecalibaculum* for the Dox group). Taken together, the above analyses suggested that the correlation structure of the microbial community in the Dox group was distinctly different from that of the Con group.

### Correlations Between Significantly Different Species and Clinical Factors in Two Groups of Mice

**Figure 5:**
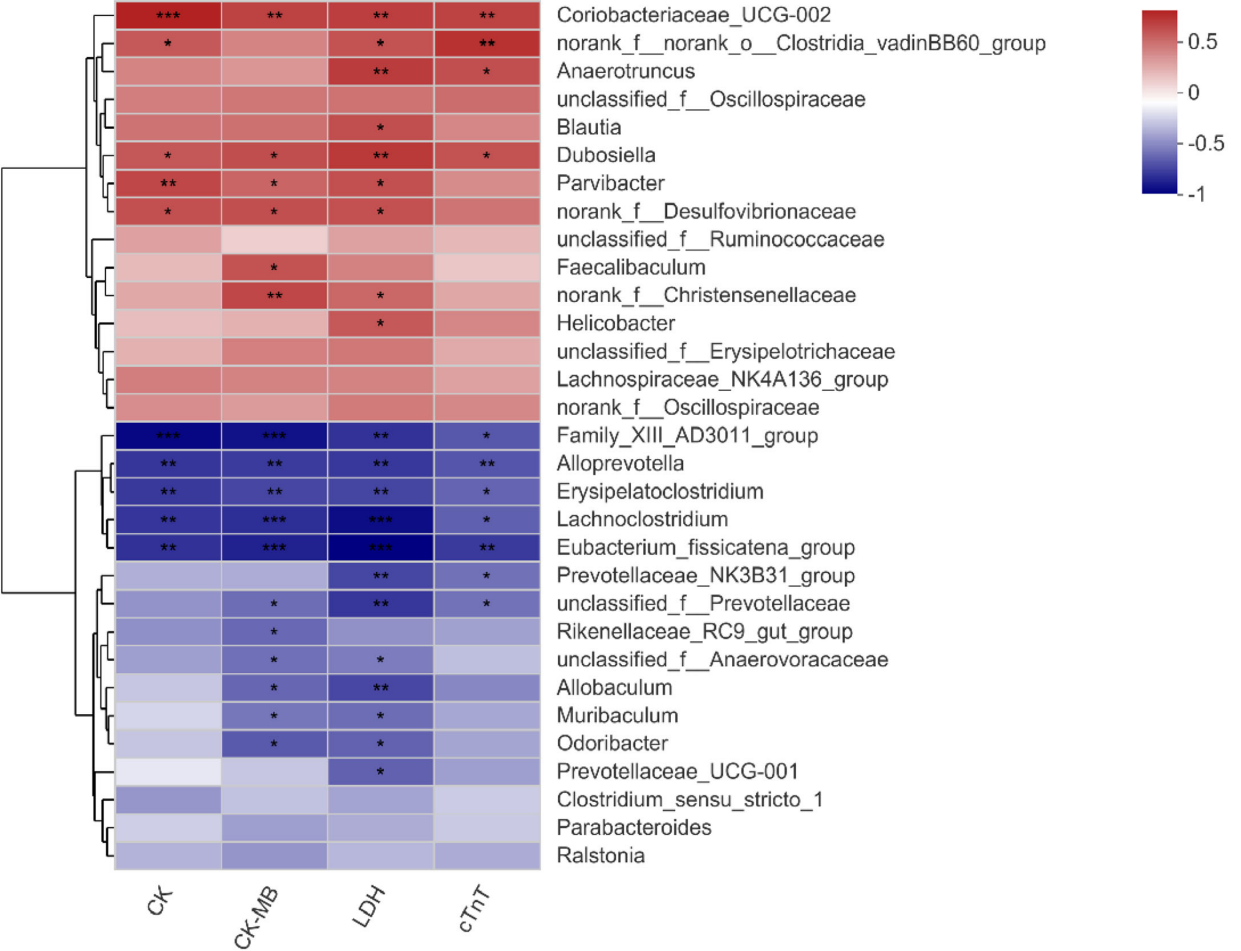
Correlation heatmap of significantly different genera and clinical factors. The x- and y-axes are clinical factors and genera, respectively. R in different colors is shown; the right side of the legend is the color range of different R values. CK, creatine kinase; CK-MB, creatine kinase-MB; LDH, lactic dehydrogenase; cTnT, cardiac troponin T. n = 10. ^*^ *p* < 0.05, ^**^ *p* < 0.01, ^***^ *p* < 0.001.

Spearman’s correlation heatmap showed that the relationship between significantly different species (**Figure 3E**) and serum biochemical parameters (CK, CK-MB, LDH, and cTnT) was different. Bacterial genera enriched in the Dox group were positively correlated with these clinical factors, while those enriched in the normal mice showed a negative correlation. Among them, Coriobacteriaceae_UCG-002 and *Dubosiella* had significant positive correlations with all the clinical parameters, and Family_XIII_AD3011_group, *Alloprevotella*, Erysipelatoclostridium, *Lachnoclostridium*, and Eubacterium_fissicatena_group showed extremely significant negative correlations with all these biochemical parameters (**Figure 5**). Therefore, it is speculated that these different species may be involved in the process of cardiotoxicity.

### Alternations of the Microbial Functional Profiles Were Revealed by Metagenomic Analysis

In this study, we used metagenomic sequencing analysis of the gut microbiomes to investigate the differences in the microbial functional composition between the Dox-treated and Con mice. Genomic DNA from the fecal specimens was extracted to obtain a total of 506,191,910 clean reads resulting in 2,646,123 contigs. A total of 4,092,442 ORFs predicted from the contigs were used for functional annotation in the COG and KEGG databases.

**Figure 6:**
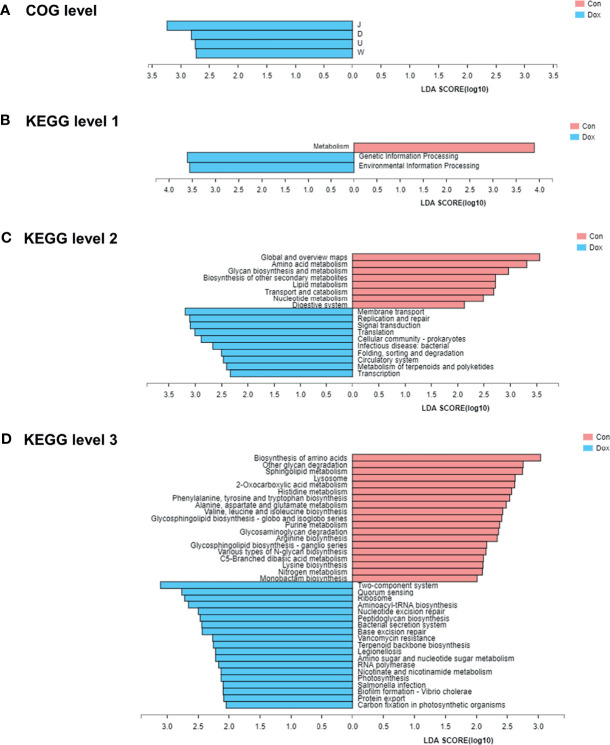
The functional pathway comparisons in metagenome between Con and Dox groups were analyzed by LEfSe analysis (LDA > 2.0, *p* < 0.05). **(A)** Histogram of the LDA scores for the differences of COG functional categories. **(B–D)** Histograms of the LDA scores for the differences of KEGG functional pathways at three levels. Con, control; Dox, doxorubicin; LDA, linear discriminant analysis; LEfSe, linear discriminant analysis effect size; COG, Cluster of orthologous groups of proteins; KEGG, Kyoto Encyclopedia of Genes and Genomes. n = 6.

To identify protein function annotation, COG analysis was performed using LEfSe analysis between the Con and Dox mice. Based on the threshold LDA values >2.0 and *p* < 0.05, we identified 4 functional COG categories that showed high enrichment in the Dox group, which were related to the translation, ribosomal structure, and biogenesis [J]; cell cycle control, cell division, and chromosome partitioning [D]; intracellular trafficking, secretion, and vesicular transport [U]; and extracellular structures [W] (**Figure 6A**). These functions could be classified into two categories: information storage and processing (Function J) and cellular processes and signaling (Functions D, U, and W). The remaining COG categories have no biologically significant differences. Overall, the cluster of cellular processes and signaling was the predominant COG category associated with the Dox mice.

To further explore the functions of differentially expressed genes, the KEGG pathways were analyzed also by LEfSe analysis (LDA > 2.0, *p* < 0.05). At KEGG level 1 (**Figure 6B**), the LEfSe bar showed that metabolism was the dominant signaling pathway in the Con group, and genetic information processing and environmental information processing were significantly enriched in the Dox group. At KEGG level 2 (**Figure 6C**), 10 differential KEGG pathways (including membrane transport, replication and repair, signal transduction, and others) were identified in the gut microbiome of Dox-treated mice, while 8 KEGG pathways (including global and overview maps, amino acid metabolism, glycan biosynthesis, and metabolism and others) were significantly increased in the Con mice. At KEGG level 3 (**Figure 6D**), we found a total of 38 statistically different functional KEGG pathways between the two groups. Half of these functions were highly enriched in the Dox group, including two-component system, quorum sensing, and ribosome. In contrast, the KEGG functions of the Con mice were enriched in the biosynthesis of amino acids, other glycan degradation, sphingolipid metabolism, and more. Thus, the Con mice and Dox mice represented completely different multiple functional pathways in the gut microbiome.

**Figure 7:**
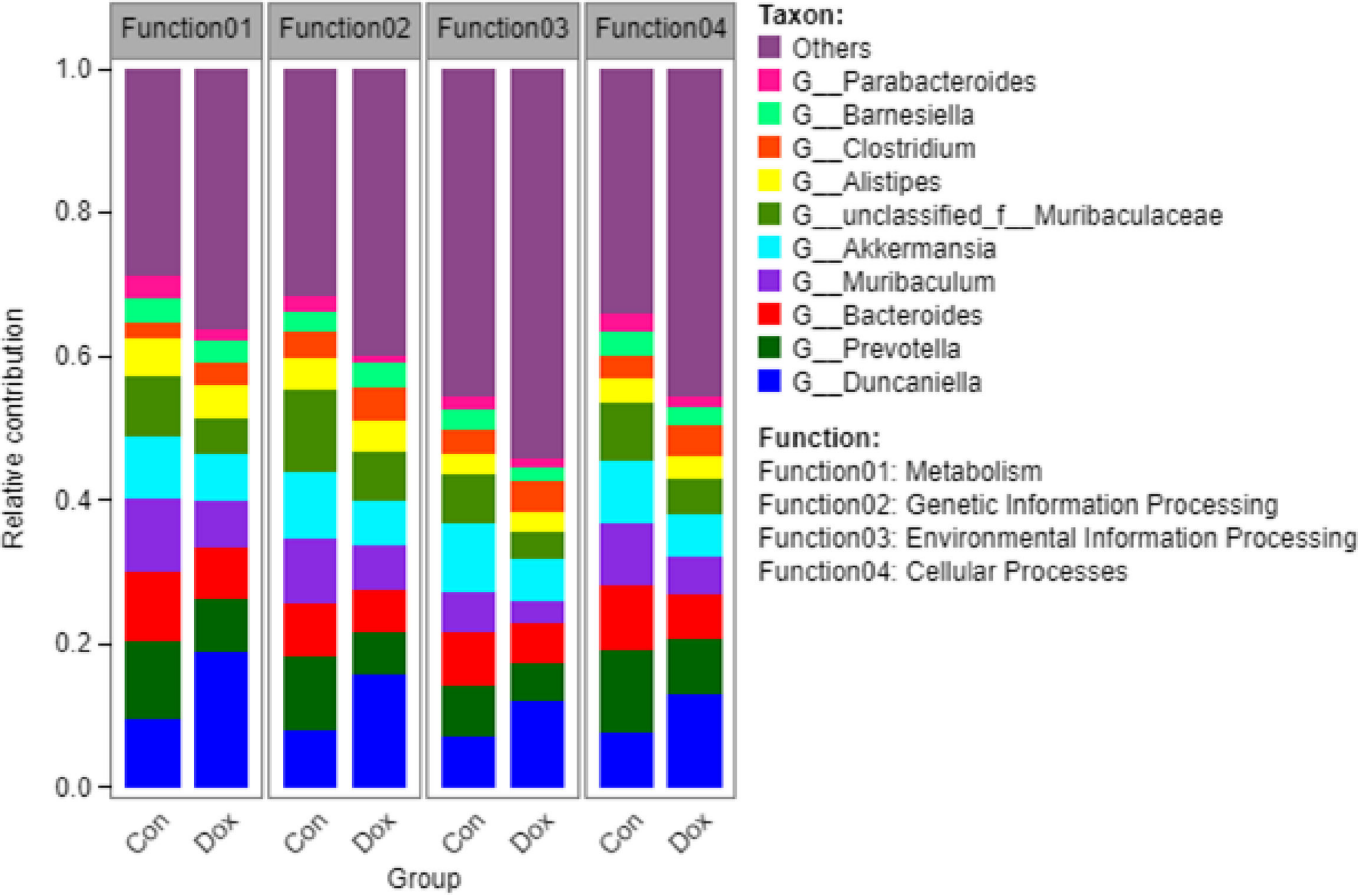
Histogram of the main species composition and their functional contribution at KEGG level 1 in Con and Dox groups. The top 10 genera and the top 4 functions are displayed. Con, control; Dox, doxorubicin; KEGG, Kyoto Encyclopedia of Genes and Genomes. n = 6.

Furthermore, to visualize the association between the gut microbiome and functional properties, we determined the top ten genera that mainly contributed to differences at KEGG level 1 pathways between the Con and Dox mice (**Figure 7**). The main functions involved in these species were metabolism, genetic information processing, environmental information processing, and cellular processes. G_Duncaniella was the main contributor of these functions and contributed significantly more to the Dox samples than the Con samples. A reduced contribution by taxa belonging to G_Prevotella and G_*Bacteroides* was also observed.

### Correlations Between Microbial Functions and Clinical Factors in the Two Groups of Mice

**Figure 8:**
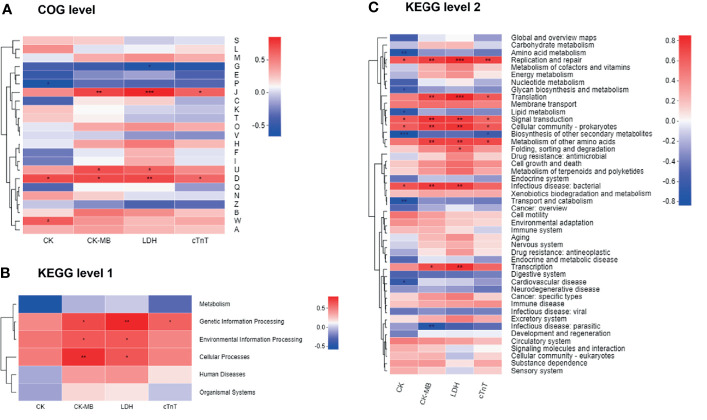
Correlation heatmaps of functional pathways and clinical factors. **(A)** Heatmap at COG level. **(B, C)** Heatmaps at KEGG level. The x- and y-axes are clinical factors and functional terms. R in different colors is shown; the right side of the legend is the color range of different R values. CK, creatine kinase; CK-MB, creatine kinase-MB; LDH, lactic dehydrogenase; cTnT, cardiac troponin T; COG, Cluster of orthologous groups of proteins; KEGG, Kyoto Encyclopedia of Genes and Genomes. n = 6. ^*^ *p* < 0.05, ^**^ *p* < 0.01, ^***^ *p* < 0.001.

We next combined the bacterial functions and serum biochemical parameters into annotated heatmaps that provided several insights into the correlation between the microbial functional profiles and DIC (**Figure 8**). At the COG level (**Figure 8A**), carbohydrate transport and metabolism [G] showed a negative significant correlation with LDH level. Inorganic ion transport and metabolism [P] revealed a significant negative correlation with the CK level. Translation, ribosomal structure, and biogenesis [J] were significantly positively correlated with CK-MB, LDH, and cTnT levels. Intracellular trafficking, secretion, and vesicular transport [U] demonstrated a significant positive correlation with CK-MB and LDH levels. Cell cycle control, cell division, and chromosome partitioning [D] were significantly positively correlated with all the biochemical factors. Extracellular structures [W] were significantly positively correlated with the CK level. Notably, the COG categories including J, U, D, and W were enriched in the Dox group (**Figure 6A**). At KEGG level 1 (**Figure 8B**), the metabolism pathway tended to be negatively, but not significantly, correlated with these biochemical factors. Genetic information processing showed a significant positive correlation with CK-MB, LDH, and cTnT levels. Environmental information processing and cellular processes were significantly positively correlated with CK-MB and LDH levels. At KEGG level 2 (**Figure 8C**), several pathways enriched in the Con group such as amino acid metabolism, glycan biosynthesis, and metabolism and lipid metabolism were negatively correlated with the clinical factors, while several pathways enriched in the Dox group such as replication and repair, translation, and signal transduction were positively correlated with the clinical factors. Collectively, gut microbiota dysfunction may be at least partially related to the DIC.

### Depletion of Gut Microbiota Attenuated Doxorubicin-Induced Cardiotoxicity

**Figure 9:**
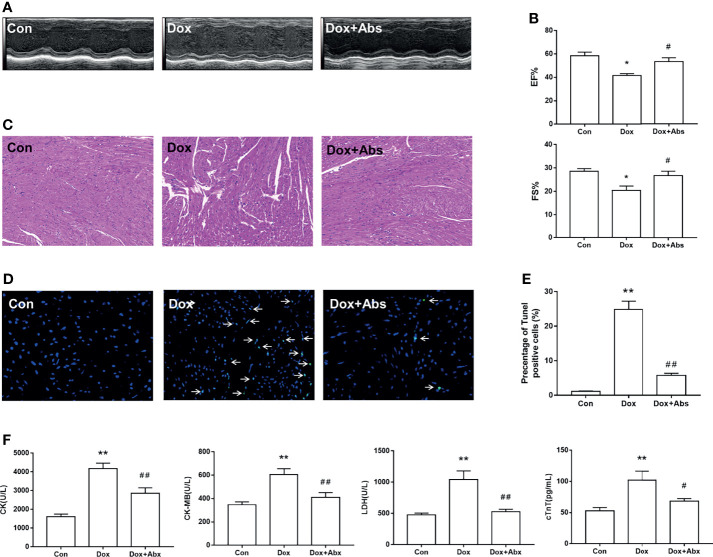
The effect of Abs on the Dox-induced cardiac dysfunction and histological injury. **(A)** M-mode echocardiograms showing Abs treatment attenuated the Dox-induced left ventricular dilation. **(B)** The parameters of left ventricular ejection fraction (EF%) and left ventricular fractional shortening (FS%). **(C)** H&E staining reflected the histological changes, magnification ×200. **(D)** Representative image of TUNEL staining, magnification ×400; white arrows indicate the apoptotic cells. **(E)** Statistical result of TUNEL staining. **(F)** The serum level of creatine kinase (CK), creatine kinase-MB (CK-MB), lactic dehydrogenase (LDH), and cardiac troponin T (cTnT). Con, control; Dox, doxorubicin; Abs, antibiotics. Data are mean ± SEM. n = 6–10. ^*^ *p* < 0.05, ^**^ *p* < 0.01 *vs.* Con; ^#^ *p* < 0.05, ^##^ *p* < 0.01 *vs.* Dox.

Next, we depleted the gut microbiota with a cocktail of Abs in Dox-treated mice to determine whether the progression of DIC is related to gut microbiota dysbiosis. Results displayed that Abs treatment attenuated the LV dilation (**Figure 9A**) and the decrease of EF% and FS% caused by Dox injection (**Figure 9B**). H&E staining results showed that inflammatory infiltrations and disorder of cardiac fiber arrangement in Dox-treated mice, while Abs administration could ameliorate this myocardial damage (**Figure 9C**). We also found that the Dox-induced cardiomyocyte apoptosis was ameliorated by treatment with Abs (**Figures 9D, E**). Moreover, Abs inhibited the effects of Dox on the serum levels of myocardial enzymes including CK, CK-MB, LDH, and cTnT (**Figure 9F**). These data suggested that depletion of the gut microbiota using a cocktail of Abs could alleviate Dox-induced myocardial injury and cardiomyocyte apoptosis.

## Discussion

The gut microbiota has been shown to have a greater impact on multiple diseases including CVDs (Kasahara and Rey, 2019) and the therapeutic effects of drugs (Tarasiuk and Fichna, 2019). This study firstly integrated the 16S rRNA gene and metagenomic sequencing to explore the association between DIC and the gut microbiota. Sequencing information not only can identify bacteria at different taxonomic levels but also can obtain functional information on the microbiome. In the current study, the mice with Dox treatment had markedly different structural compositions and functional networks on the gut microbiota as compared with the normal mice. Moreover, the cardiomyocyte apoptosis and myocardial damage caused by Dox could be suppressed by depleting the gut microbiota. Therefore, it is reasonable for us to propose that the gut microbiota and their functions, at least in part, contribute to DIC development.

According to the results of microbial alpha diversity analysis, the Chao index showed a statistical difference with a downward trend in Dox-treated mice. Another research on a rat model indicated that Dox significantly decreased the species diversity of fecal bacteria, which was consistent with our results. However, the relative abundance of Firmicutes-to-Bacteroidetes ratio, contrary to the trend of this research, was decreased in Dox-treated rats (Wu et al., 2019). *Lactobacillus*, one of the Firmicutes bacteria, is frequently either positively or negatively related to human disease and chronic conditions (Heeney et al., 2018; Slattery et al., 2019). Cisplatin was found to decrease the relative abundance of *Lactobacillus* in the fecal bacterial community, and supplementation with *Lactobacillus* could prevent cisplatin-induced cardiotoxicity (Zhao et al., 2018). In contrast to our study using Dox-treated mice, we observed a slight increase in the proportion of this bacterium, but there was no significant difference compared to the Con mice. Given the conflicting reports, intestinal *Lactobacillus* level and its role in DIC need further investigation.

The correlation network analysis also revealed the disorder of gut microbiota structure in Dox-treatment mice, which performed fewer relationships but more complex networks. It should be noted that the harmful bacterium *Bacteroides* was chosen as one of the putative keystone genera in the Dox network. It is an obligate anaerobic, gram-negative rod-shaped bacterium that is usually symbiotic and a common opportunistic pathogen in clinical infections (Rocha and Smith, 2013). A 16S rRNA sequencing study showed that, compared with the no-treatment Wistar rats, Dox treatment caused intestinal flora disorder, increasing the harmful flora *Bacteroides fragilis* (Zhao et al., 2021).

In line with our bacterial difference analysis, Liu et al. reported that phylum Actinobacteriota becomes abundant in the Dox mice (Liu et al., 2020b), suggesting that Actinobacteriota may be a minus factor in the cardiotoxicity process. A previous study demonstrated that the combination of multi-walled carbon nanotubes with Dox increased the abundance of family Coriobacteriaceae within the phylum Actinobacteria in mice, polarized colonic macrophages to an M1-like pro-inflammatory phenotype, and thus upregulated proinflammatory factors TNF-α and IL-1β in DIC (Liu et al., 2020b). Here, we found that genus Coriobacteriaceae_UCG-002 not only significantly increased in the Dox group but also positively correlated with the serum levels of myocardial enzymes. Further experiment at this genus level seemed to be necessary. Genus *Dubosiella* is a member of short-chain fatty acid (SCFA) producers (Mao et al., 2019; Bojovic et al., 2020). A previous study showed that chlorogenic acid increased the abundance of *Dubosiella* and improved metabolic endotoxemia (Ye et al., 2021). In contrast, the protective effects of yellow wine polyphenolic compounds were associated with a lower abundance of *Dubosiella* in Dox-treated rats (Lin et al., 2021). Here, we demonstrated that *Dubosiella* increased in the Dox-treated mice and positively correlated with all the myocardial enzyme levels. Further experiments are being carried out to investigate the role of these microbes (e.g., Coriobacteriaceae_UCG-002, *Dubosiella*, Family_XIII_AD3011_group, and *Alloprevotella*) in DIC.

At the functional level with metagenomic sequencing, we used COG and KEGG analyses to annotate the functional discrimination of the gut microbiota between the two groups. For COG functional annotation, cellular processes and signaling were the predominant categories associated with the Dox mice, which included cell cycle control, cell division, intracellular trafficking, secretion, vesicular transport, and extracellular structures. The KEGG pathway analysis further acknowledged the gut microbiota functions might contribute to DIC pathogenesis through cellular processes, such as membrane transport, replication and repair, and signal transduction. Correlation heatmaps also revealed those cellular processes were significantly positively correlated with the clinical factors. Extracellular structures of Gram-negative bacteria contain an endotoxin called lipopolysaccharide (LPS) (Cheng et al., 2018). Cancer chemotherapy, such as Dox, can induce intestinal mucositis and damage (Kaczmarek et al., 2012). Thus, LPS can enter the bloodstream through the impaired intestinal barrier and lead to the expression of a wide array of inflammatory downstream products (such as tumor necrosis factor (TNF), IL-1, and IL-6) *via* the toll-like receptor 4 (TLR4) pattern recognition receptor (Lu et al., 2008; Tang et al., 2019). Dysregulation of the cellular structure and function of the microbiota may lead to increased LPS transport, which is involved in the process of cardiotoxicity. Edematous patients with chronic HF were also found to have higher blood levels of endotoxin and cytokines (Niebauer et al., 1999). Therefore, it can be inferred that the alleviation of DIC after depletion of the gut microbiota may be attributed to the lower endotoxin levels.

Moreover, our functional enrichment analysis revealed obvious variation of metabolism processes such as amino acid metabolism, glycan biosynthesis and metabolism, lipid metabolism, and other secondary metabolites between the two groups of mice. The correlation heatmaps at the KEGG level also demonstrated the relationship between altered metabolic functions of the gut microbiota and DIC. Accumulating evidence has suggested that gut microbial metabolites, including bile acids, SCFAs, trimethylamine *N*-oxide (TMAO), and amino acid metabolites are mechanistically linked to the pathogenesis of CVD (Mamic et al., 2021). Bile acids can activate the bile acid receptor (known as FXR) and G-protein-coupled receptors (Tang et al., 2019). FXR modulates metabolism and inflammation and is involved in myocardial apoptosis and fibrosis (Calkin and Tontonoz, 2012; Pu et al., 2013). Several SCFAs exert anti-inflammatory effects through regulatory T-cell activation to mitigate cardiac hypertrophy and fibrosis (Bartolomaeus et al., 2019). Butyric acid is beneficial to the DIC models, and its derivative phenylalanine-butyramide could reduce Dox cardiotoxicity in human cellular models, thereby attenuating Dox-induced reactive oxygen species production (Russo et al., 2019). TMAO is a gut microbiota-dependent metabolite of specific dietary nutrients, which is mainly produced from the bacterial phyla Firmicutes and Actinobacteria (Romano et al., 2015). DOX-induced cardiac fibrosis could be aggravated by TMAO through activation of the NLRP3 inflammasome (Li et al., 2019). Here, we found that the proportions of Firmicutes and Actinobacteria had an upward trend in the Dox mice compared with the Con mice. These findings facilitate our understanding of Dox-related cardiac changes or diseases. However, further research needs to be conducted for exploring the link between metabolites of the gut microbiome and DIC.

It has been reported that the heart is a priority target for Dox toxicity. However, this anticancer drug also damages other organs like the brain, kidney, and liver (Carvalho et al., 2009). For example, Dox administration can induce the decline of cognitive function (Jansen et al., 2008) and liver injury (Greupink et al., 2006). An experiment in a high-sugar and high-fat diet model showed that the abundance of Coriobacteriaceae involved in cholesterol metabolism was increased, and the altered gut microbiota and their metabolites resulted in systemic impacts on both hepatic metabolism and cognitive function (Jena et al., 2020). In our study, Coriobacteriaceae level was also found to be elevated in the Dox mice and positively correlated with clinical factors. We speculate that those increased bacteria in the Dox group were not only associated with cardiac toxicity but may also affect other comorbidities induced by Dox.

This study proved the correlations between the composition/function of the gut microbiota and DIC in the mouse model, but further rigorous experimental models depleted or colonized with a specific microbiota should be performed to identify the key bacteria. Even though several biological functions and pathways appear involved in the DIC process have been explained, we still need to further clarify these results of the biometric analysis by molecular signaling experiments. Furthermore, metabolomics and metatranscriptomics are ultimately required to explore the changes in the levels of metabolites of the gut microbiome and understand the metabolism mechanism of DIC.

## Conclusion

Taken together, our results demonstrated that Dox modified the composition and function of the gut microbiome in mice. We provide important information that supports that the gut microbiota promotes DIC partially through influencing cell processes and biochemical metabolism. The gut microbiota might be a vital participant in a potential therapeutic strategy to attenuate the cardiotoxicity of chemotherapeutic drugs.

## Data Availability Statement

The datasets presented in this study can be found in online repositories. The names of the repository/repositories and accession number(s) can be found in the article/**Supplementary Material**.

## Ethics Statement

The animal study was reviewed and approved by the Medicine Animal Welfare Committee of Xiangya School of Medicine (SYXK-2015/0017).

## Author Contributions

BZ and WL conceived and designed the experiments. JH, SW, CJ, and ZX performed the experiments. JH, JL, and WP analyzed the data. JH, BZ, and WL wrote the paper. All authors contributed to the article and approved the submitted version.

## Funding

This study was supported by grants from the National Natural Scientific Foundation of China (Nos. 82173911 and 81973406), Fundamental Research Funds for the Central Universities of Central South University (No. 2021zzts1057), Hunan Provincial Natural Scientific Foundation (Nos. 2019JJ50849 and 2020JJ4823), Scientific Research Project of Hunan Provincial Health and Family Planning Commission (No. 202113050843), and Bethune Quest-Pharmaceutical Research Capacity Building Project (No. B-19-H-20200622).

## Conflict of Interest

The authors declare that the research was conducted in the absence of any commercial or financial relationships that could be construed as a potential conflict of interest.

## Publisher’s Note

All claims expressed in this article are solely those of the authors and do not necessarily represent those of their affiliated organizations, or those of the publisher, the editors and the reviewers. Any product that may be evaluated in this article, or claim that may be made by its manufacturer, is not guaranteed or endorsed by the publisher.
